# Optimized Primer for Partial N and F Gene Sequencing of PPRV

**DOI:** 10.3390/pathogens15101065

**Published:** 2026-10-08

**Authors:** Milovan Milovanović, Christian Korthase, Klaas Dietze, Bernd Hoffmann

**Affiliations:** Friedrich-Loeffler-Institut, 17493 Greifswald-Insel Riems, Germany; mmsetter12@gmail.com (M.M.); christian.korthase@googlemail.com (C.K.); klaas.dietze@fli.de (K.D.)

**Keywords:** peste des petits ruminants virus, *Morbillivirus caprinae*, molecular diagnostics, RT-PCR, F gene, N gene, genetic characterization, sequencing

## Abstract

Despite global efforts to eradicate Peste des petits ruminants (PPR), this disease remains one of the most devastating viral infections affecting small domestic ruminants. Sensitive and specific molecular diagnostic tools are essential components of successful eradication strategies. Molecular diagnostics and genetic characterization of Peste des petits ruminants virus (PPRV) are commonly based on partial sequencing of the PPRV fusion (F) and nucleocapsid (N) genes, but the primers currently in use were developed several decades ago. In this study, we have developed and validated new primer sets for partial amplification of the PPRV F- and N-genes. PPRV isolates representing more recently circulating representatives of all four viral lineages were included. A total of 16 primer pairs, including previously published primers, were evaluated for their performance in partial amplification of the PPRV F and N genes. The primer pairs PPRV-F-K9 (460 bp) and PPRV-N-K15 (408 bp) demonstrated the highest sensitivity across all four lineages. Additionally, two alternative primer pairs, PPRV-F-K6 (399 bp) and PPRV-N-K3 (366 bp), exhibited comparable sensitivity, making them reliable alternatives for partial F and N gene sequencing. Overall, the newly developed primer pairs exhibited improved sensitivity compared with previously published primers, supporting their suitability for enhanced molecular diagnostics and genetic characterisation of PPRV.

## 1. Introduction

Peste des petits ruminants (PPR) is one of the most economically significant viral diseases affecting small domestic ruminants in Africa, the Middle East, Asia [1], and Europe [2]. It is caused by the Peste des petits ruminants virus (PPRV), which is a single-stranded, non-segmented, negative-sense RNA virus belonging to the *Morbillivirus caprinae* species [3]. The transboundary nature of PPRV spread, together with the disease’s severe clinical manifestations and high morbidity and mortality rates, particularly in naïve herds, has drawn considerable international attention. Consequently, PPR is listed as a notifiable animal disease by the World Organisation for Animal Health (WOAH). In collaboration with the Food and Agriculture Organization of the United Nations (FAO), it has been targeted for global eradication by 2030 [4].

The genetic characterization of PPRV, based on partial genome sequencing of the nucleocapsid (N) and the fusion glycoprotein (F) genes, has revealed the existence of four distinct genetic lineages within a single serotype [5,6]. Partial N- and F-gene sequencing has formed the basis of PPRV molecular epidemiology for more than two decades and has enabled the classification of PPRV isolates into four major genetic lineages and the comparison of viruses originating from geographically and temporally distant outbreaks. Numerous epidemiological and phylogenetic studies have subsequently relied on these established sequence regions, generating a large body of comparable sequence data that continues to be used for lineage assignment and investigation of virus circulation and transboundary spread [1,7,8]. The geographic distribution of these lineages indicates that lineages I and II are restricted to West Africa, lineage III is predominantly found in East Africa and the Middle East, and lineage IV circulates widely across Asia, the Middle East, Africa, [1,9] and Europe [2]. The lineage of PPRV has been shown to be more accurately determined when targeting the N gene, which exhibits greater genetic variability and higher divergence between lineages than the highly conserved F gene. This increased variability is particularly evident in regions III and IV of the N gene, which contain less conserved sequences [10,11,12].

As the primers most commonly used for partial genome sequencing of PPRV have been in use for over two decades, sequence variation accumulated in the targeted regions may compromise their performance for some recently circulating PPRV strains. In the present study, we have developed and validated a novel set of primers for partial amplification of the PPRV F and N genes across all four genetic lineages.

## 2. Materials and Methods

### 2.1. PPRV Sample Selection

This study used PPRV cell culture isolates belonging to all four genetic lines, covering older reference strains as well as more recently isolated strains from different regions (Table 1). Complete genome information was available for all ten PPRV isolates, which were then assigned to a genetic line based on this data.

### 2.2. RNA Extraction and Real-Time RT-PCR

The IndiMag Pathogen Kit (Indical Bioscience, Leipzig, Germany) was used to extract viral nucleic acid from PPRV cell culture isolates on the semi-automated IndiMag 48 platform according to the manufacturer’s instructions. The conserved region of the nucleocapsid protein was amplified using a specific primer–probe mix [13], with a heterologous control system [14] simultaneously detected via the HEX channel. Detailed information on the method used is described in Milovanović et al. [15].

Ct values ranging from 14.1 to 23.5 were determined for all PPRV cell culture isolates using RT-qPCR. To avoid unrealistically high viral genome loads in the samples during optimisation of the sequencing primers, dilutions of the extracted viral RNA were prepared. PPRV-negative pooled RNA/DNA from goats and sheep was used for this purpose. The viral genome load of all PPRV strains included in the study was adjusted to a moderate level (Ct values between 26.5 and 30.2). The viral RNA dilutions used for primer preselection are listed in the figure captions. The RT-qPCR results for the starting and diluted materials, with β-actin detection, are shown in the Supplementary Materials (Figure S1).

### 2.3. RT-PCR for Partial Amplification of the N and F Gene of PPRV

The SuperScript™ III One-Step RT-PCR System with Platinum™ Taq DNA Polymerase (Invitrogen, Carlsbad, CA, USA) was used for conventional gel-based PCR to partially amplify the N- and F-genes of PPRV. The following were mixed: 0.75 µL nuclease-free water, 6.25 µL 2× reaction mix, 0.5 µL enzyme mix, 0.5 µL MgSO_4_, 1 µL of each the various combinations of forward and reverse primers (concentration 10 pmol/µL), and 2.5 µL RNA template. RT-PCR was performed using a C1000 PCR cycler (Bio-Rad, Hercules, CA, USA) with the following cycle conditions: a reverse transcriptase reaction for 15 min at 50 °C; Taq polymerase activation for 2 min at 95 °C; and then 45 cycles consisting of denaturation for 15 s at 95 °C, primer annealing for 20 s at 60 °C, and extension for 35 s at 72 °C. This was followed by a final extension of 5 min at 72 °C. To visualise the PCR product, a standard horizontal electrophoresis was performed using a 1.5% agarose gel stained with ethidium bromide. A 100bp DNA marker was used to confirm the size of the PCR product.

### 2.4. Sanger Sequencing Procedure

The corresponding PPRV N and F bands were excised from the agarose gel and the amplified DNA was purified using the QIAquick Gel Extraction Kit (Qiagen, Hilden, Germany) according to the manufacturer’s instructions. Sanger sequencing of the PCR products was performed using the LightRun service from Eurofins Genomics (Ebersberg, Germany). For this purpose, 5 µL of the purified amplicon was mixed with 5 µL of primer (5 pmol/µL), barcoded and sent to Eurofins for further sequencing. Each PCR amplicon was sequenced using both the forward and reverse primers. The resulting raw data were analysed using Geneious Prime v2021.0.1 (Biomatters Ltd., Auckland, New Zealand) to generate consensus sequences for the PPRV N- and F-genes.

## 3. Results

### 3.1. In Silico Selection of Primer for Partial N- and F-Gene Sequencing

The Geneious Prime v2021.0.1 software (Biomatters Ltd., Auckland, New Zealand) was used to design the primers to detect the PPRV-F and PPRV-N genes. A total of 585 PPRV N-gene and 498 PPRV F-gene sequences, belonging to all four available lineages, were obtained from the National Centre for Biotechnology Information (NCBI) in January 2023. Care was taken to ensure that those regions of the N- and F-genes were used that are typically amplified with the standard primer pairs described by Couacy-Hymann in 2002 [5] and by Forsyth and Barrett in 1995 [6]. This is the only way to perform phylogenetic analyses that combine both the long-established and the newly generated partial sequences of N and F.

Based on the multiple alignment of the available N and F gene sequences, three forward and reverse primers were selected for each gene to enable sensitive amplification. In addition to these, the newly selected primers, the previously published primers for the N- and F-genes described by Couacy-Hymann (2002) [5] and by Forsyth and Barrett (1995) [6] were also tested for comparison. All primers considered in the study are listed in Table 2.

### 3.2. Preselection of Primer for Partial N- and F-Gene Sequencing

After selecting the primers in silico, a preselection process was conducted in which all available forward and reverse primers were combined to identify the optimal combination. The 16 primer combinations for the N-gene yielded fragments ranging in size from 351 to 486 base pairs, whereas the F primers used yielded amplification products ranging from 332 to 487 bp. Detailed information on the combinations of forward and reverse primers can be found in Tables S1 and S2 of the Supplementary Material.

To further narrow down the suitable primer combinations for sensitive amplification of the PPRV-F and PPRV-N genes, a 100-fold dilution of the cell culture virus PPRV/UAE2021/V346.2 (Ct value approximately 28), which belongs to lineage IV (GenBank accession number OM867572), was used. PPRV-negative goat RNA, extracted from EDTA-treated blood, was used to dilute the viral cell culture RNA. This diluted viral RNA, which included the nucleic acid from the goat, was used to test the sixteen different combinations of forward and reverse primers for amplification of the N- and F-genes (PPR-N-K1 to -K16 and PPR-F-K1 to -K16, see Supplementary Material, Tables S1 and S2).

#### 3.2.1. Preselection of Primers for the PPRV-N Gene

When the results obtained using all 16 different combinations of forward and reverse primers for the PPRV-N gene were evaluated, the primers PPR-N-K12 and PPR-N-K13 failed to amplify the 360 bp and 486 bp target regions of PPRV, respectively. Other primer combinations were able to amplify the target region of the PPRV-N gene, with the expected length of the PCR products being observed during agarose gel electrophoresis (Figure 1). Based on the size and signal intensity of the observed PCR products, the four best primer combinations were selected for further analysis: PPR-N-K1, PPR-N-K3, PPR-N-K14, and PPR-N-K15.

#### 3.2.2. Preselection of Primers for the PPRV-F Gene

Of the sixteen different primer combinations used to detect the PPRV-F protein gene, the PPR-F-K3 primer was the only one to amplify the target region. The expected 372 bp PCR product was not observed during agarose gel electrophoresis. All other primer combinations were able to amplify the target region of the PPRV-F gene. This resulted in the detection of a PCR product of the expected size using agarose gel electrophoresis (Figure 2). Of all the combinations used, the primers PPR-F-K5, PPR-F-K6, PPR-F-K9, and PPR-F-K11 produced the best results, as determined by the size and intensity of the PCR product in agarose gel electrophoresis. Figure 2 shows the results for all sixteen different primer combinations targeting the PPRV-F gene of PPRV/UAE2021/V346.2.2.

### 3.3. Performance of Preselected Primers for the Partial Amplification of All Four PPRV Lineages

The four primer combinations selected for the N-gene and the four primer combinations preferred for the F-gene were used to amplify partial sequences of various PPRV isolates from all four genetic lineages. Based on the band intensity and quality observed during gel electrophoresis, the most suitable primer combinations for partial amplification of the N- and F-genes were further narrowed down. Table 1 lists the PPRV isolates used for these analyses, and the Supplementary Materials contain detailed results in Figures S2–S7.

Of all the strains tested from the four genetic lineages, the primer combination PPR-N-K15, which amplified a 408 bp fragment, demonstrated the greatest capacity for partial amplification of the PPRV-N gene. Not only did this primer combination produce the strongest specific band in gel electrophoresis, it also showed no signs of non-specific amplification. The second-best primer combination was PPR-N-K3, which successfully generated a 366 bp fragment of the PPRV-N gene from all tested PPRV strains. However, in certain cases, it exhibited slightly lower fragment intensity that the PPR-N-K15 primer combination. During agarose gel electrophoresis of the other two pre-selected primer combinations (PPR-N-K1 and PPR-N-K14), a generally lower fragment intensity was observed. However, these two primer combinations were also able to amplify 444 bp and 418 bp fragments, respectively, from all tested PPRV strains. Upon closer examination, agarose gel electrophoresis revealed very a low intensity of the PCR fragments generated by the primer combination PPR-N-K1 in strains V135.2 and V407.2 (PPRV of lineage IV), as well as by the primer combination PPR-N-K14 in strains V135.2 and V284.1 (PPRV of lineages IV and I, respectively).

Examination of the results of agarose gel electrophoresis of the amplified PPRV-F gene revealed that the PPR-F-K9 primer produced the best results, amplifying a 460 bp fragment of the PPRV-F gene. Slightly lower success was observed when using the PPR-F-K6 primer to amplify a 399 bp fragment of the PPRV-F gene. In this case, the intensity of the bands in the agarose gel electrophoresis was lower for certain genetic lines than for the PPR-F-K9 primer. The other two preselected primers, PPR-F-K5 and PPR-F-K11, also successfully amplified all four PPRV genetic lines, yielding corresponding fragments of 451 bp and 401 bp, respectively. However, a lower PCR amplicon intensity was occasionally observed in the gel for these two primer combinations.

### 3.4. Analytical Sensitivity and Specificity of the Final Primer Combinations Selected for Partial Amplification of the PPRV-F and PPRV-N Genes

The two most effective primer combinations for partial amplification of the PPRV-F gene (PPR-F-K6 and PPR-F-K9) and the PPRV-N gene (PPR-N-K3 and PPR-N-K15) were used to amplify log10 dilution series of representative PPRV strains from all four genetic lineages (V284.3, V285.1, V286.2, and V288.3). The corresponding semi-quantitative viral genome loads were determined by RT-qPCR for all dilution series (Figure 3). Identical viral RNA dilution series were used to determine the analytical sensitivity of the two preferred primer combinations for partial amplification of the F and N genes. According to the results of agarose gel electrophoresis, the primer combinations PPR-F-K9 and PPR-N-K15 proved to be the most sensitive. Two other primer combinations, PPR-F-K6 and PPR-N-K3, proved to be slightly less sensitive but still yielded sufficiently good results for all four PPRV strains, even with weakly positive samples. Based on the results of the second-choice primers, it was determined in two cases that the band intensity did not correspond to the virus dilution (Figure 4A, lane 6 and Figure 4C, lane 4 and 5). This effect can be multifactorial and could be related to the concentration of template and residual inhibitors, which can affect PCR efficiency, as well as to variations introduced during serial dilution, PCR setup or gel loading. The results of the agarose gel electrophoresis are shown in Figure 4.

The results presented here clearly show that partial amplification of the N and F genes of various PPRV strains is possible with very high sensitivity using the optimized primer combinations. Despite the larger PCR products, gel-based sequencing RT-PCR performs well compared to the results of real-time RT-PCR, the standard method for diagnosis, and achieves nearly comparable analytical sensitivities.

Analytical specificity against other closely related morbilliviruses, such as rinderpest virus, canine distemper virus or measles virus, as well as against all sequences deposited in GenBank, was assessed in silico. For 7 of the 8 preferred primers, no homology whatsoever could be detected with morbilliviruses or other ruminant pathogens. Only the primer PPRV-N-1680R from the PPRV-N-K15 primer set showed a 100 per cent match with N-gene sequences of the rinderpest virus. As the forward primer PPRV-N-1273F, which is part of the PPRV-N-K15 primer set, showed no significant homology with the rinderpest virus genome, corresponding amplification of the PCR product is highly unlikely. Due to the destruction of rinderpest virus RNA that has taken place, this assumption cannot be verified. In this context, however, it must also be emphasized that the rinderpest virus has been eradicated worldwide, and that other closely related morbilliviruses, such as the distemper virus or the measles virus, are highly host-specific. Consequently, these viruses are not expected to be detected in species susceptible to PPRV.

### 3.5. Evaluation of the Sanger Sequencing Procedure

In a final test, we investigated whether the four preferred primer combinations for the N and F genes not only produced an amplification product of the appropriate size but also whether these PCR products yielded chromatograms that could be analysed using the Sanger sequencing method. To this end, viral RNA from strain PPRV/UGA2020/V407.2 was amplified at a 100-fold dilution using the four preferred primer combinations for the N- and F-genes (see Figure S7). After separating the PCR amplicons using an agarose gel, the corresponding bands were excised and purified. The respective PCR products were sequenced in both directions using the homologous forward and reverse primers (Table 2, Tables S1 and S2). The first-choice primer pairs PPRV-F-K9 (460 bp; PPRV-F-5717F and PPRV-F-6176R) and PPRV-N-K15 (408 bp; PPRV-N-1273F and PPRV-N-1680R), as well as the second-choice primer pairs, PPRV-F-K6 (399 bp; PPRV-F-5769F and PPRV-F-6167R) and PPRV-N-K3 (366 bp; PPRV-N-1273F and PPRV-NP4-1638R), yielded high-quality sequence chromatograms for the PPRV-F and PPRV-N genes, respectively. Based on the individual sequences of different orientations, consensus sequences of the expected length could be generated at any time. Examples of chromatograms for the four preferred primer combinations for partial sequencing of the N and F genes have been included in the Supplementary Material as Figures S8–S11. No mutations from the expected sequence or ambiguous results were identified during analysis of the raw sequence data. The final BLASTN (version 2.16.0+) analysis of the PPRV-F and -N genes confirmed that the obtained sequences showed the highest similarity to PPRV lineage III sequences.

## 4. Discussion

Reliable and sensitive molecular diagnostic methods are essential for the detection and molecular characterization of PPRV, which should be one of the main pillars in disease control and eradication. While clinical signs can support the initial suspicion of PPR, they are not pathognomonic and require laboratory confirmation. Basic molecular methods such as RT-PCR remain an important diagnostic approach, particularly in settings where more technically demanding methods are not readily available. Despite implementation of global control efforts, PPR has continued to spread. In the publication of Imanbayeva et al. [16], critical gaps in PPR control were identified by analyzing the eradication progress. Next to diagnostic potential, partial PPRV-N and PPRV-F gene sequencing provide good insight via molecular characterization of PPRV, allowing us to better understand the epidemiological origin of the detected viruses. A recent example is the emergence of PPRV in Greece in 2024. By partial N-gene sequence analysis it was confirmed that the virus belongs to PPRV lineage IV circulating in North-East Africa [17].

Most of the primers currently used for partial sequencing and gel-based RT-PCR diagnostics of the PPRV-F and -N genes target the conserved region of the PPRV-F gene and the least conserved region of the PPRV-N gene [5,6]. The continued relevance of partial N-gene sequencing is also reflected in subsequent molecular epidemiological studies and reviews. Comparative analyses of N- and F-gene sequences have demonstrated that the N gene generally provides greater phylogenetic resolution than the F gene, and N-gene sequencing has therefore become the preferred approach for lineage assignment and molecular epidemiological investigations of newly detected PPRV isolates [1,8,18]. However, although the primers published by Forsyth and Barrett [6] target a well-conserved region of the PPRV-F gene, they were unable to detect the partial F gene of PPRV lineage IV (V346.2) in our prescreening procedure, as the 372 bp product was not observed in agarose gel electrophoresis. One possible explanation is that these primers were developed three decades ago and that the F gene to be amplified originated from a recent PPRV outbreak in Barbary sheep [15]. Conversely, although the PPRV-N gene primers published by Couacy-Hymann et al. [5] were able to amplify the corresponding partial N gene of this strain, the signal intensity in agarose gel electrophoresis was not satisfactory.

In addition, a large number of new complete PPRV sequences have been published in recent years. In silico assessment of the primer binding sites for the primer combinations published by Couacy-Hymann et al. [5] and Forsyth and Barrett [6] shows that some mismatches exist. Consequently, reduced sensitivity of these published and long-used primer combinations is to be expected when amplifying the corresponding N and F genes in certain PPRV strains. This concern is supported by previous observations that, although the established NP3/NP4 primer pair remains widely used and performs well with high-quality samples, amplification and subsequent sequencing may become more challenging when viral RNA concentrations are low or when field samples are of reduced quality [19]. This highlights the need for continuously updated primer systems that retain compatibility with historically established sequence regions while improving amplification performance across genetically diverse PPRV strains. For this reason, we selected an additional 15 N primer pair and an additional 15 F primer pair for comparison and tested them in this study. Importantly, the continued use of these established primer regions has resulted in a large body of comparable partial N- and F-gene sequences that forms the basis of many published phylogenetic and molecular epidemiological studies. Maintaining compatibility with these historical datasets therefore allows newly generated sequence data to be integrated into existing datasets for long-term monitoring of PPRV evolution, lineage distribution and transboundary virus spread [20,21,22,23,24]. Therefore, in the present study, we specifically selected primer combinations that not only improved amplification sensitivity but also retained the genomic regions covered by the long-established primer pairs described by Couacy-Hymann et al. [5] and Forsyth and Barrett [6].

The primer pairs ultimately selected for the partial PPRV-F and PPRV-N genes were found to have high analytical sensitivity, as demonstrated by their ability to amplify highly diluted PPRV samples from all four genetic lineages. Furthermore, the high specificity of the selected primers was confirmed by the correct band sizes observed in the agarose gel and the final sequencing results. Additionally, primer specificity was supported by the absence of non-specific amplification in the presence of PPRV-negative sheep or goat RNA used as background in the viral dilution series. Taken together, these findings indicate that the optimized primer pairs can improve the generation of reliable partial N- and F-gene sequence data for downstream phylogenetic and molecular epidemiological analyses.

## 5. Conclusions

Molecular detection methods with high sensitivity and specificity are crucial for the successful global eradication of PPR. Therefore, an update to the molecular diagnostic methods for partial sequencing of the PPRV N- and F-genes was warranted based on currently available genetic information. In this study, using a limited number of samples available, the primer combinations PPRV-N-K15 and PPRV-F-K9 were identified as the most sensitive, and thus the most suitable for initial testing, of all primer pairs tested. The primer pairs PPRV-N-K3 and PPRV-F-K6 also produced good results, making them a good second choice for the sensitive and specific detection of partial PPRV-N and PPRV-F gene fragments from all known PPRV strains. Using alternative primer combinations to successfully amplify the target gene increases diagnostic reliability and offers advantages in the characterization of novel PPRV strains. The selected primer pairs combine improved amplification sensitivity with compatibility with established sequence regions, facilitating the continued use of partial N- and F-gene sequencing for PPRV molecular, epidemiological, and phylogenetic analyses. The performance of the primer sets should be further investigated using various sample matrices from clinically affected animals.

## Figures and Tables

**Figure 1:**
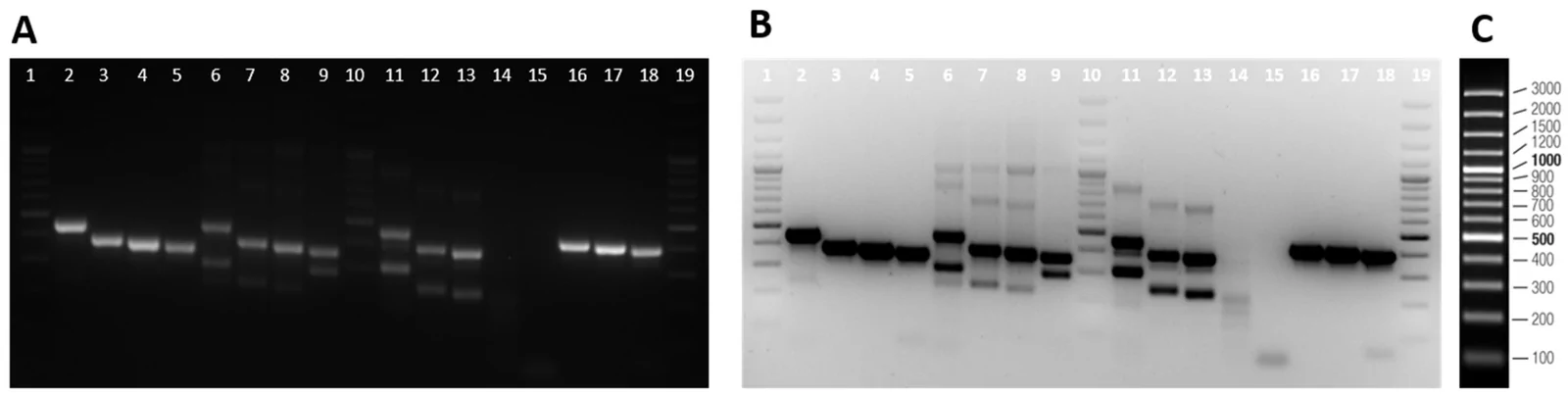
Results of agar gel electrophoresis of an RT-PCR assay for the N gene using a 100-fold dilution of RNA from PPRV/UAE2021/V346.2/LineageIV. (**A**) (standard) and (**B**) (inverted): from left to right, positions 1, 10, and 19: Gene Ruler 100 bp marker; positions from 2 to 9—PPR-N-K1 (444 bp), PPR-N-K2 (376 bp), PPR-N-K3 (366 bp), PPR-N-K4 (351 bp), PPR-N-K5 (446 bp), PPR-N-K6 (378 bp), PPR-N-K7 (368 bp), and PPR-N-K8 (353 bp) respectively; positions from 11 to 18, PPR-N-K9 (453 bp), PPR-N-K10 (385 bp), PPR-N-K11 (375 bp), PPR-N-K12 (360 bp), PPR-N-K13 (486 bp), PPR-N-K14 (418 bp), PPR-N-K15 (408 bp), and PPR-N-K16 (393 bp) respectively; (**C**): Gene Ruler 100 bp marker.

**Figure 2:**
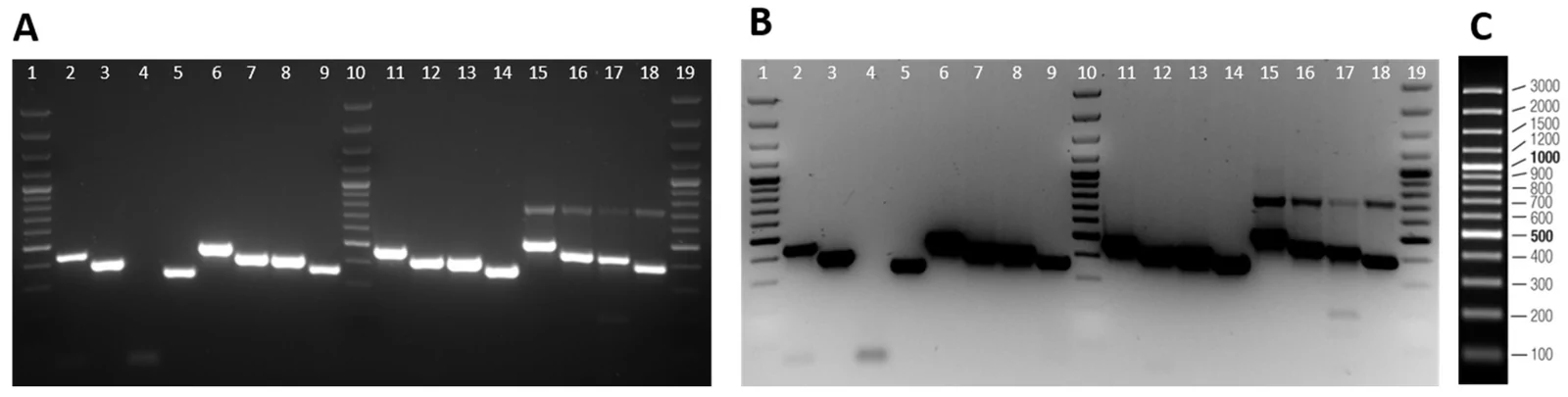
Results of agar gel electrophoresis of an RT-PCR assay for the F gene using a 100-fold dilution of RNA from PPRV/UAE2021/V346.2/LineageIV. (**A**) (standard) and (**B**) (inverted): from left to right, positions 1, 10, and 19: Gene Ruler 100 bp marker; positions from 2 to 8—PPR-F-K1 (431 bp), PPR-F-K2 (379 bp), PPR-F-K3 (372 bp), PPR-F-K4 (332 bp), PPR-F-K5 (451 bp), PPR-F-K6 (399 bp), PPR-F-K7 (392 bp), and PPR-F-K8 (352 bp) respectively; positions from 11 to 18, PPR-F-K9 (460 bp), PPR-F-K10 (408 bp), PPR-F-K11 (401 bp), PPR-F-K12 (361 bp), PPR-F-K13 (487 bp), PPR-F-K14 (435 bp), PPR-F-K15 (428 bp), and PPR-F-K16 (388 bp) respectively; (**C**): Gene Ruler 100 bp marker.

**Figure 3:**
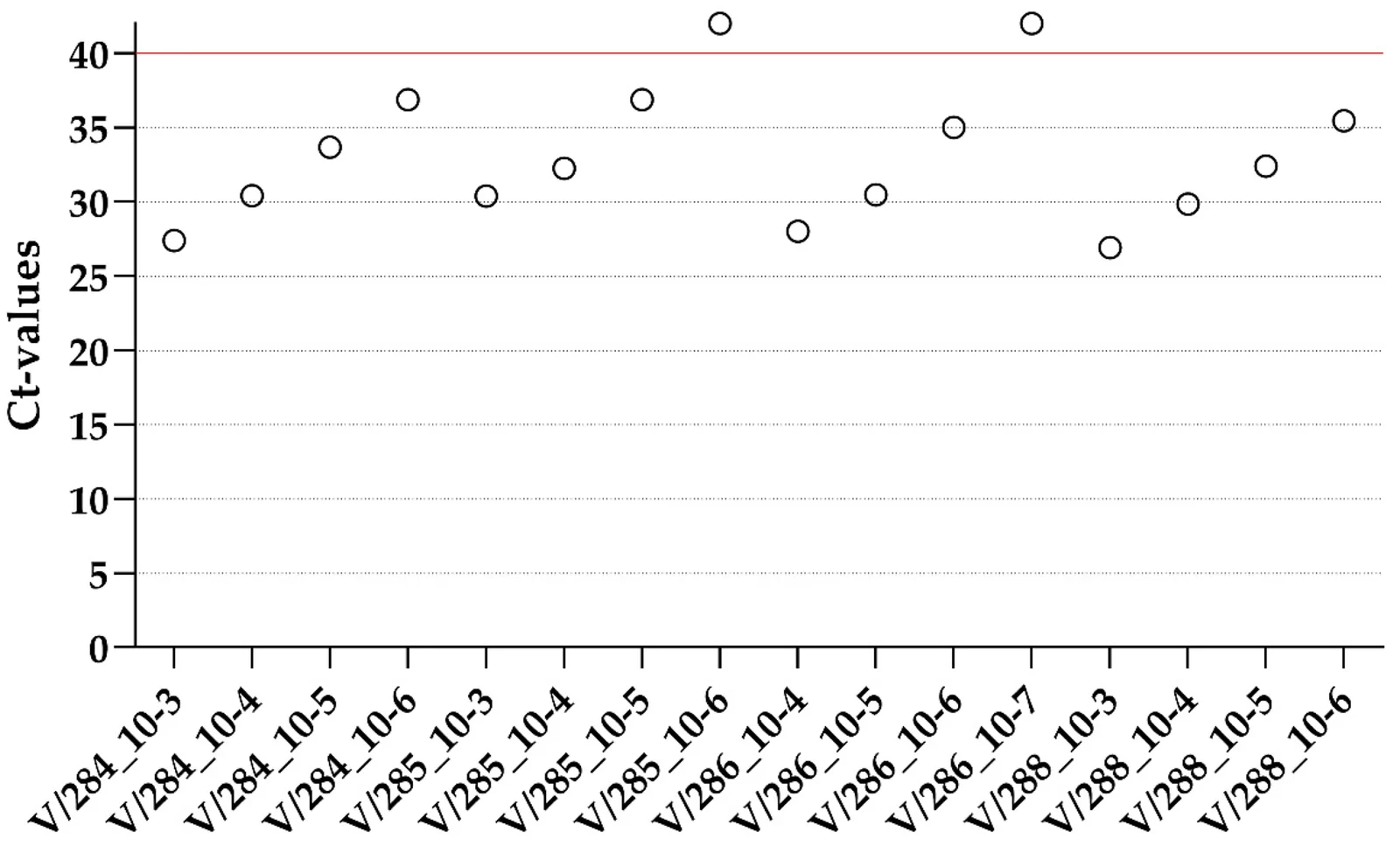
RT-qPCR results for log_10_ dilution series of representative PPRV strains from all four genetic lineages (Lineage I—V284, Lineage III—V285, Lineage II—V286, and Lineage IV—V288). Threshold for the red line: Ct value > 40—negative; 40 ≤ Ct value—positive.

**Figure 4:**
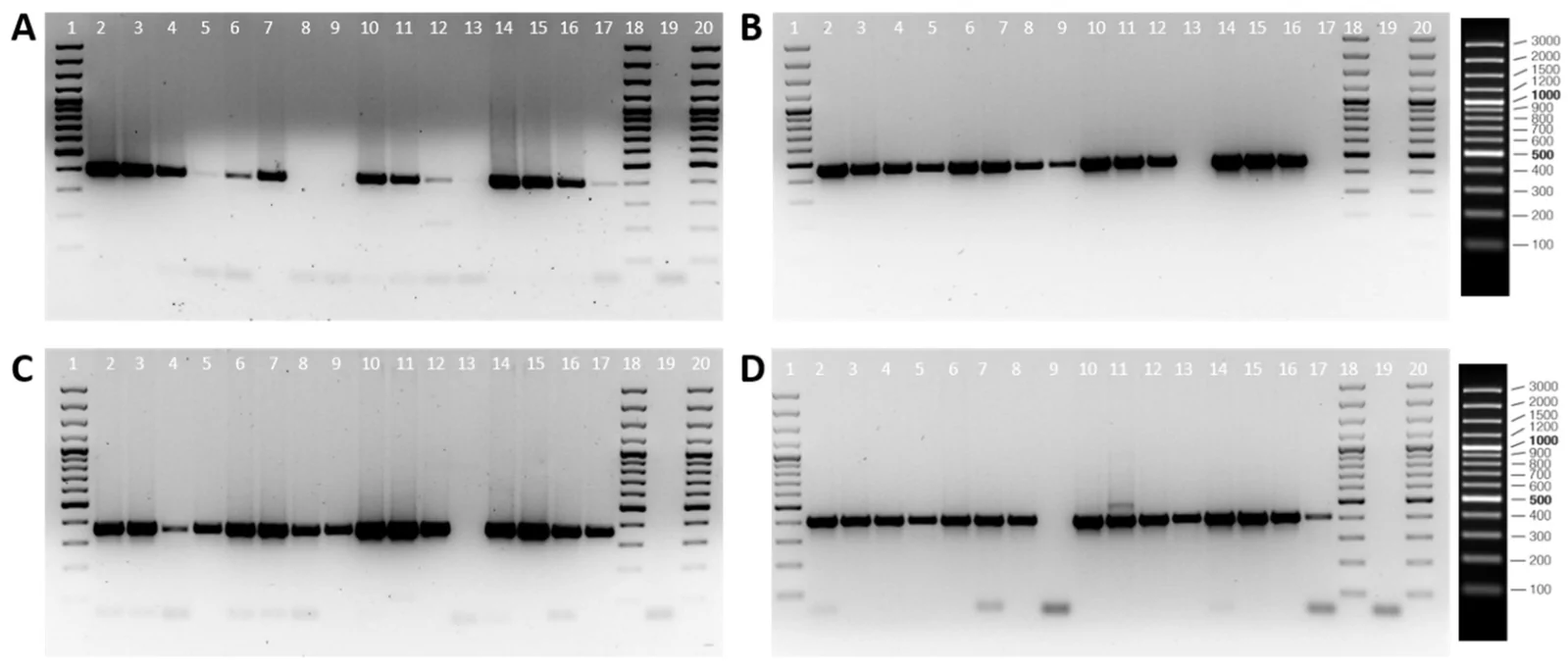
Results of agarose gel electrophoresis of conventional RT-PCR. (**A**–**D**)—positions 1, 18, and 20: Gene Ruler 100 bp marker; positions 2 through 5—V248.1 Line I (dilutions 10^−3^–10^−6^ each); positions 6 through 9—V285.1 Line III (10^−3^–10^−6^ each); positions 10 through 13—V286.2 Line II (10^−4^–10^−7^ each); positions 14 through 17—V288.3 Line IV (10^−3^ to 10^−6^ each); and position 19—Negative control (RNase-free water). (**A**,**B**)—Partial amplification of the PPRV F gene using the primers PPR-F-K6 (399 bp) and PPR-F-K9 (460 bp), respectively; (**C**,**D**)—Partial amplification of the PPRV-N gene using the primers PPR-N-K3 (366 bp) and PPR-N-K15 (408 bp), respectively.

**Table 1:** List of the PPRV used in this study.

PPRV Strain	Internal PPRV Number	PPRV Lineage	Original Host	NCBI GenBank Accession Number
PPRV/UAE2018	V135.2	IV	Sand gazelle	MN369542
PPRV/IND2013	V242.2	IV	Goat	MN369543
PPRV/UAE2019	V283.3	IV	Sand gazelle	unpublished
PPRV/ICV89	V284.3	I	Goat	EU267273
PPRV/Sudan1972	V285.3	III	Goat	OR286505.1
PPRV/NIG75/1	V286.2	II	Goat	X74443.2
PPRV/Kurdistan2011	V288.3	IV	Bezoar ibex	MK408669
PPRV/UAE2021-1	V345.3	IV	Sand gazelle	unpublished
PPRV/UAE2021-2	V346.2; V346.3	IV	Barbary sheep	OM867572
PPRV/UGA2020	V407.2	III	Goat	PP188031

**Table 2:** List of the forward and reverse primers used for the partial amplification of PPRV-N and PPRV-F genes.

Target	Name/Position	Sequence (5′-3′)	Autor
PPRV-N	PPRV-N-1195F	GTC TCG GAC AGG AGA TGG T	This study
	PPRV-N-1263F	TCA CAG CAG AGG AAG CYA AAC T	This study
	PPRV-N-1273F	GAA GCY AAA CTR GTC TCG GAA AT	This study
	PPRV-NP3-1288F	TCT CGG AAA TCG CCT CAC AGA CTG	[5]
	PPRV-NP4-1638R	CCT CCT CCT GGT CCT CCA GAA TCT	[5]
	PPRV-N-1640R	TCC CTC CTC YTG GYC CTC	This study
	PPRV-N-1647R	GTC TTC TCC CTC CTC YTG G	This study
	PPRV-N-1680R	TGA GGA GAT CCT TGT CGT TGT	This study
PPRV-F	PPRV-F-5717F	ATA TAA CRG CCA TCR ACA ATT GTA C	This study
	PPRV-F-5769F	ATT GCT RAT CAC AGT GTT AAA RCC T	This study
	PPRV-F1-5776F	ATC ACA GTG TTA AAG CCT GTA GAG G	[6]
	PPRV-F-5815F	ATA ACC AAG AAT GTA AGA CCA ATT CA	This study
	PPRV-F2-6147R	GAG ACT GAG TTT GTG ACC TAC AAG C	[6]
	PPRV-F-6167R	GTG TAR TAC CTA AGG AGY TTG AG	This study
	PPRV-F-6176R	AGG ATC TCG GTR TAR TAC CTA AG	This study
	PPRV-F-6203R	TCT CGA AGR CTR GGC CCG A	This study

## Data Availability

The original contributions presented in this study are included in the article/Supplementary Material. Further inquiries can be directed to the corresponding author.

## Supplementary Materials

The following supporting information can be downloaded at https://www.mdpi.com/article/10.3390/pathogens15101065/s1, Table S1. List of forward and revers primers for partial amplification of PPRV-N gene. Table S2. List of forward and revers primers for partial amplification of PPRV-F gene. Figure S1. RT-qPCR results of PPRV cell culture isolates used in this study. Figures S2–S7. Results of gel electrophoresis using the four preferred combinations of forward and reverse primers for the partial amplification of the PPRV-F and -N genes. Figures S8–S11. Presentation of the chromatograms and the consensus sequence following the Sanger sequencing procedure for the primer combination PPRV-F-K6, PPRV-F-K9, PPRV-N-K3, and PPRV-N-K15.
