# Supplementary material for: Optimized Primer for Partial N and F Gene Sequencing of PPRV
Source: Pathogens. 2026 Oct 8;15(10):1065. doi: 10.3390/pathogens15101065 (PMC13708387; doi:10.3390/pathogens15101065)
Supplement: Supplementary file 1 [file pathogens-15-01065-s001.zip › pathogens-4571010-supplementary.pdf]

Table S1. List of forward and revers primers for partial amplification of PPRV-N gene. Yellow marked combination of the forward and revers primer with expected size of the PCR product in based pars (bp).

Name/position	Melting temperature (°C)	Primer mix name															
		PPR-N-K1	PPR-N-K2	PPR-N-K3	PPR-N-K4	PPR-N-K5	PPR-N-K6	PPR-N-K7	PPR-N-K8	PPR-N-K9	PPR-N-K10	PPR-N-K11	PPR-N-K12	PPR-N-K13	PPR-N-K14	PPR-N-K15	PPR-N-K16
PPRV-N-1195F	59.5																
PPRV-N-1263F	60.1-62.1																
PPRV-N-1273F	59.2-62.9																
PPRV-NP3-1288F	66.9																
PPRV-NP4-1638R	68.5																
PPRV-N-1640R	58.4-62.9																
PPRV-N-1647R	59.5-61.6																
PPRV-N-1680R	59.5																
**Sequence length (bp)**		**444**	**376**	**366**	**351**	**446**	**378**	**368**	**353**	**453**	**385**	**375**	**360**	**486**	**418**	**408**	**393**

Table S2. List of forward and revers primers for partial amplification of PPRV-F gene. Yellow marked combination of the forward and revers primer with expected size of the PCR product in based pars (bp).

**Name/position**	**Melting temperature (°C)**	**Primer mix name**															
		**PPR-F-K1**	**PPR-F-K2**	**PPR-F-K3**	**PPR-F-K4**	**PPR-F-K5**	**PPR-F-K6**	**PPR-F-K7**	**PPR-F-K8**	**PPR-F-K9**	**PPR-F-K10**	**PPR-F-K11**	**PPR-F-K12**	**PPR-F-K13**	**PPR-F-K14**	**PPR-F-K15**	**PPR-F-K16**
PPRV-F-5717F	59.2-62.5																
PPRV-F-5769F	59.2-62.5																
PPRV-F1-5776F	64.1																
PPRV-F-5815F	60.1																
PPRV-F2-6147R	65.8																
PPRV-F-6167R	59.2-62.9																
PPRV-F-6176R	59.2-62.9																
PPRV-F-6203R	59.5-63.6																
**Sequence length (bp)**		**431**	**379**	**372**	**332**	**451**	**399**	**392**	**352**	**460**	**408**	**401**	**361**	**487**	**435**	**428**	**388**

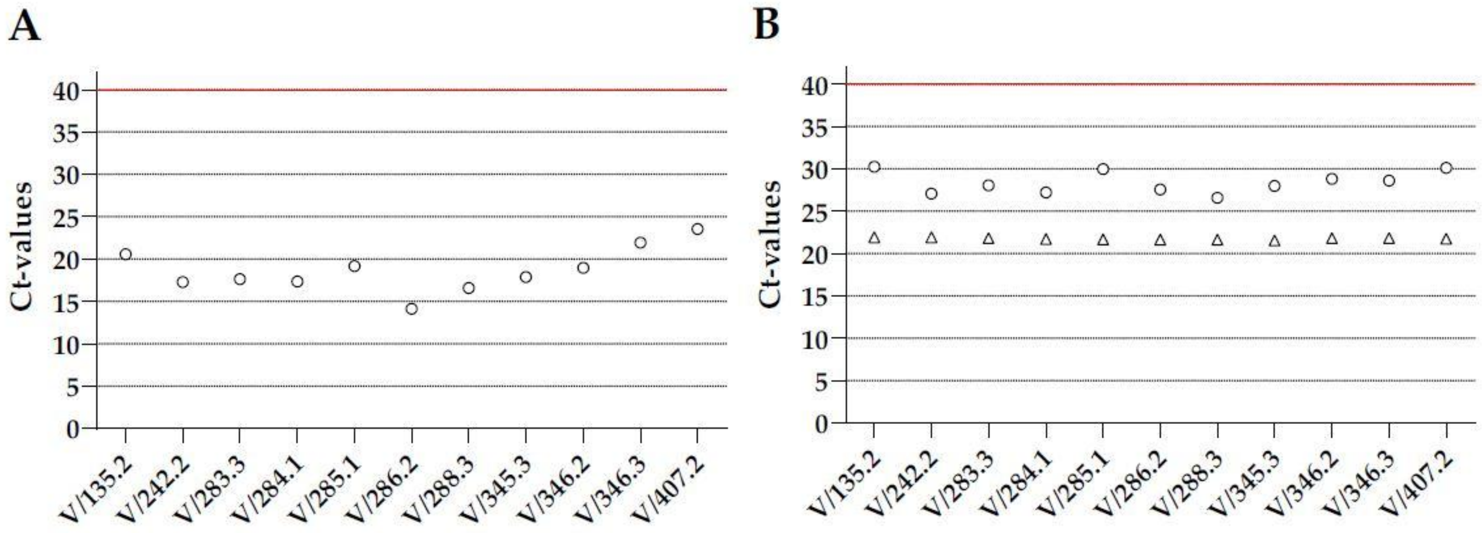

Figure S1. RT-qPCR results of PPRV cell culture isolates used in this study. Figure-A – RT-qPCR results of originally extracted PPRV from the virus bank; figure-B (circles) – RT-qPCR results of 10-log diluted extracted RNA of PPRV cell culture isolates in goat and sheep RNA; figure B (triangles) – internal control ß-actin of goat and sheep.

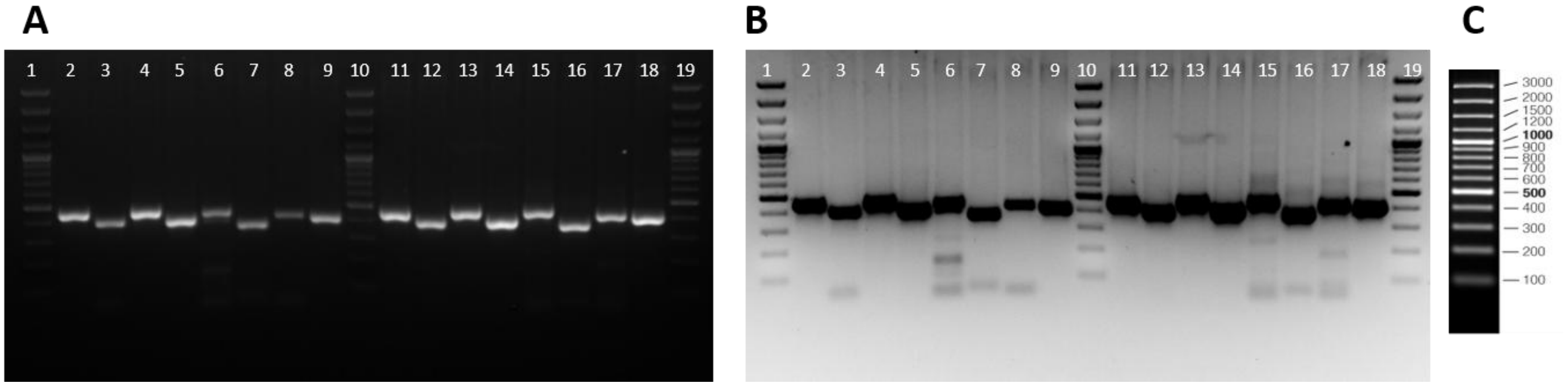

Figure S2. Results of gel electrophoresis using the four preferred combinations of forward and reverse primers for the partial amplification of the PPRV-F and -N genes. A-standard and B-inverted depiction. Positions 1, 10 and 19 Gene Ruler 100 bp marker; position from 2 to 9 – PPRV/UAE2018/V135.2/LineageIV (RNA dilution 10 to -3): PPR-F-K5, PPR-F-K6, PPR-F-K9, PPR-F-K11, PPR-N-K1, PPR-N-K3, PPR-N-K14, and PPR-N-K15, respectively; position from 11 to 18 – PPRV/IND2013/V242.2/LineageIV (RNA dilution 10 to -3): PPR-F-K5, PPR-F-K6, PPR-F-K9, PPR-F-K11, PPR-N-K1, PPR-N-K3, PPR-N-K14, and PPR-N-K15, respectively; C) Gene Ruler 100 bp marker.

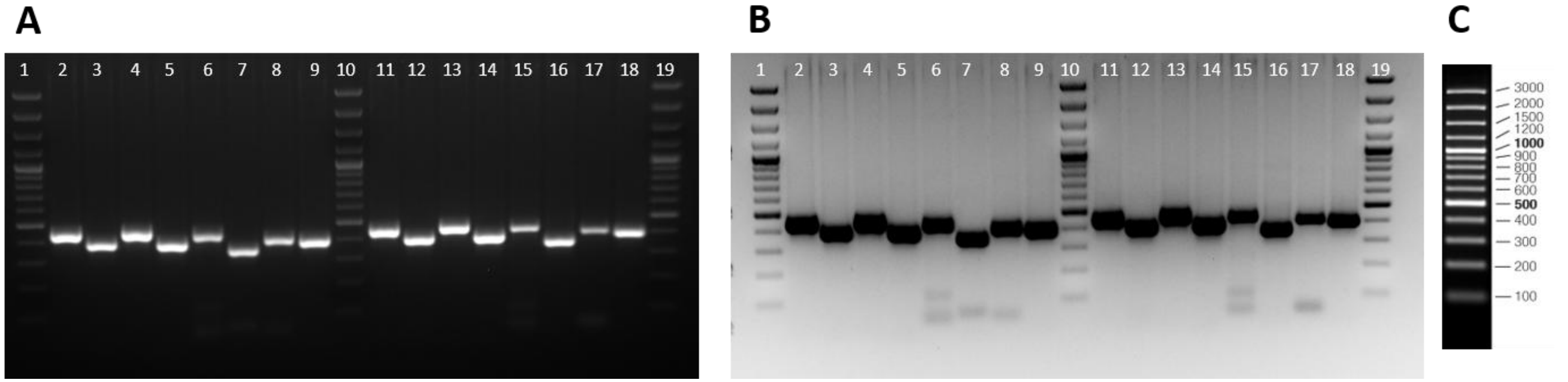

Figure S3. Results of gel electrophoresis using the four preferred combinations of forward and reverse primers for the partial amplification of the PPRV-F and -N genes. A-standard and B-inverted depiction. Positions 1, 10 and 19 Gene Ruler 100 bp marker; position from 2 to 9 – PPRV/UAE2019/V283.3/LineageIV (RNA dilution 10 to -3): PPR-F-K5, PPR-F-K6, PPR-F-K9, PPR-F-K11, PPR-N-K1, PPR-N-K3, PPR-N-K14, and PPR-N-K15, respectively; position from 11 to 18 – PPRV/ICV89/V284.3/LineageI (RNA dilution 10 to -3): PPR-F-K5, PPR-F-K6, PPR-F-K9, PPR-F-K11, PPR-N-K1, PPR-N-K3, PPR-N-K14, and PPR-N-K15, respectively; C) Gene Ruler 100 bp marker.

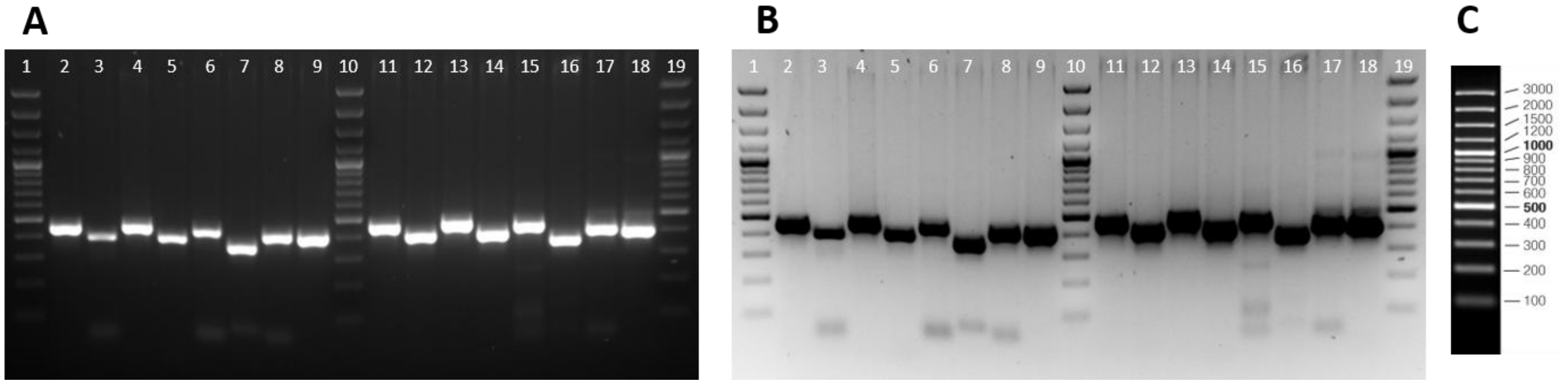

Figure S4. Results of gel electrophoresis using the four preferred combinations of forward and reverse primers for the partial amplification of the PPRV-F and -N genes. A-standard and B-inverted depiction. Positions 1, 10 and 19 Gene Ruler 100 bp marker; position from 2 to 9 – PPRV/Sudan1972/V285.3/LineageIII (RNA dilution 10 to -3): PPR-F-K5, PPR-F-K6, PPR-F-K9, PPR-F-K11, PPR-N-K1, PPR-N-K3, PPR-N-K14, and PPR-N-K15, respectively; position from 11 to 18 – PPRV/NIG75/1/V286.2/LineageII (RNA dilution 10 to -4): PPR-F-K5, PPR-F-K6, PPR-F-K9, PPR-F-K11, PPR-N-K1, PPR-N-K3, PPR-N-K14, and PPR-N-K15, respectively; C) Gene Ruler 100 bp marker.

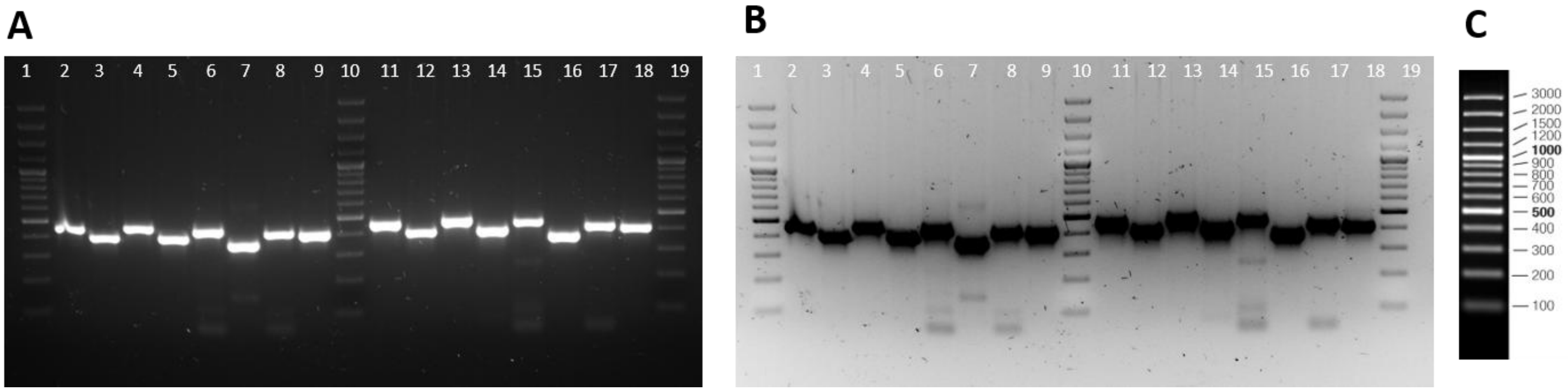

Figure S5. Results of gel electrophoresis using the four preferred combinations of forward and reverse primers for the partial amplification of the PPRV-F and -N genes. A-standard and B-inverted depiction. Positions 1, 10 and 19 Gene Ruler 100 bp marker; position from 2 to 9 – PPRV/Kurdistan2011/V288.3/LineageIV (RNA dilution 10 to -3): PPR-F-K5, PPR-F-K6, PPR-F-K9, PPR-F-K11, PPR-N-K1, PPR-N-K3, PPR-N-K14, and PPR-N-K15, respectively; position from 11 to 18 – PPRV/UAE2021-1/V345.3/LineageIV (RNA dilution 10 to -3): PPR-F-K5, PPR-F-K6, PPR-F-K9, PPR-F-K11, PPR-N-K1, PPR-N-K3, PPR-N-K14, and PPR-N-K15, respectively; C) Gene Ruler 100 bp marker.

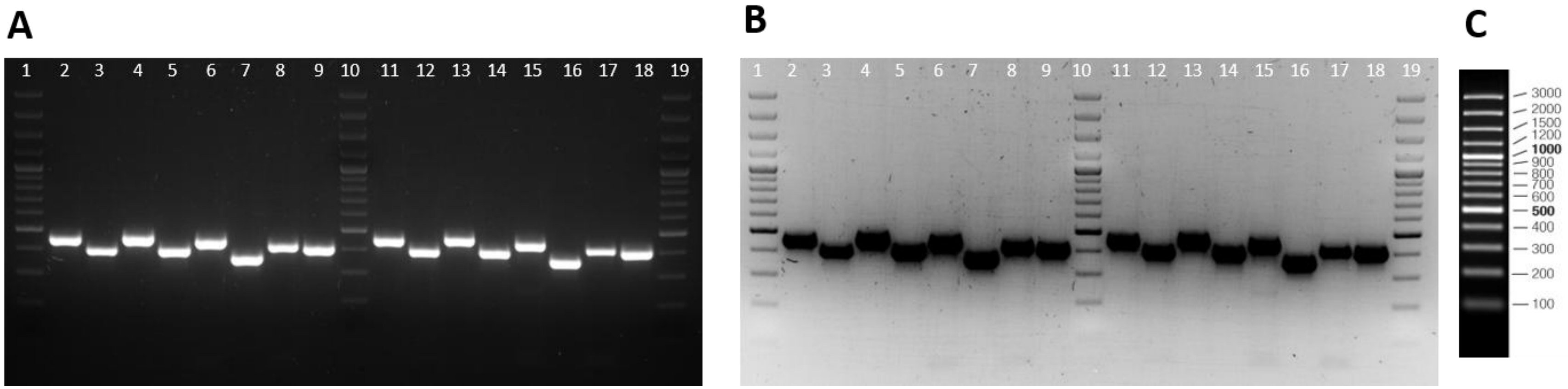

Figure S6. Results of gel electrophoresis using the four preferred combinations of forward and reverse primers for the partial amplification of the PPRV-F and -N genes. A-standard and B-inverted depiction. Positions 1, 10 and 19 Gene Ruler 100 bp marker; position from 2 to 9 – PPRV/UAE2021-2/V346.3/LineageIV (RNA dilution 10 to -3): PPR-F-K5, PPR-F-K6, PPR-F-K9, PPR-F-K11, PPR-N-K1, PPR-N-K3, PPR-N-K14, and PPR-N-K15, respectively; position from 11 to 18 – PPRV/UAE2021-2/V346.2/LineageIV (RNA dilution 10 to -2): PPR-F-K5, PPR-F-K6, PPR-F-K9, PPR-F-K11, PPR-N-K1, PPR-N-K3, PPR-N-K14, and PPR-N-K15, respectively; C) Gene Ruler 100 bp marker.

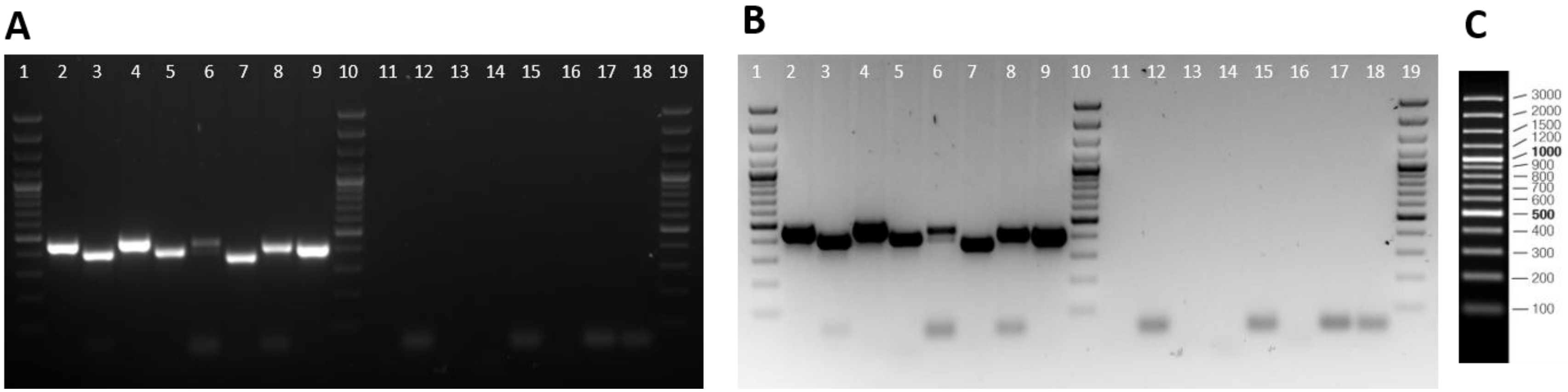

Figure S7. Results of gel electrophoresis using the four preferred combinations of forward and reverse primers for the partial amplification of the PPRV-F and -N genes. A-standard and B-inverted depiction. Positions 1, 10 and 19 Gene Ruler 100 bp marker; position from 2 to 9 – PPRV/UGA2020/V407.2/LineageIV (RNA dilution 10 to -2): PPR-F-K5, PPR-F-K6, PPR-F-K9, PPR-F-K11, PPR-N-K1, PPR-N-K3, PPR-N-K14, and PPR-N-K15, respectively; position from 11 to 18 – sheep and goat pool RNA (negative control RNA): PPR-F-K5, PPR-F-K6, PPR-F-K9, PPR-F-K11, PPR-N-K1, PPR-N-K3, PPR-N-K14, and PPR-N-K15, respectively; C) Gene Ruler 100 bp marker.

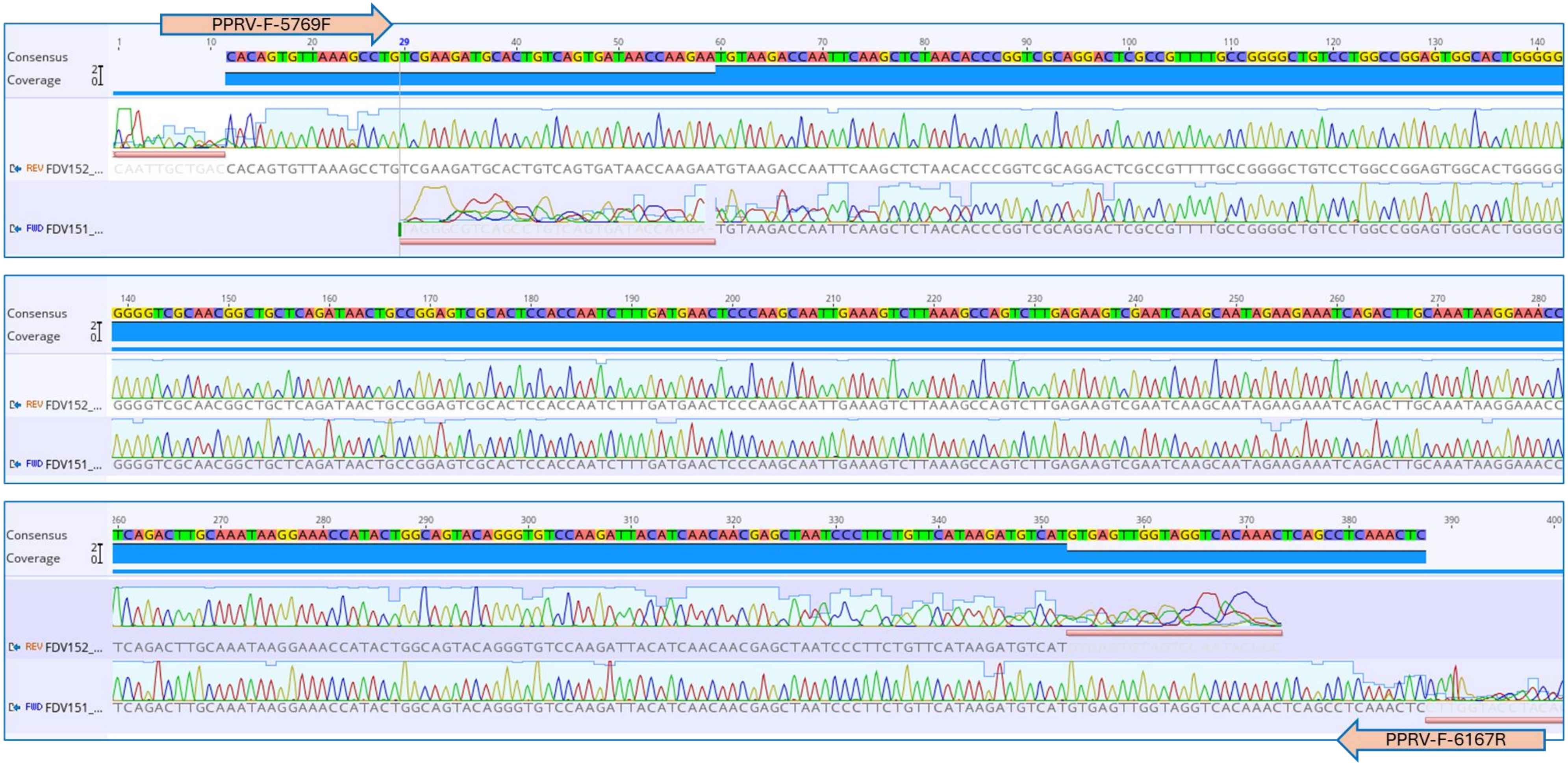

Figure S8. Primer combination PPRV-F-K6: Presentation of the chromatograms and the consensus sequence following the Sanger sequencing procedure. The positions of the primers used are indicated by arrows.

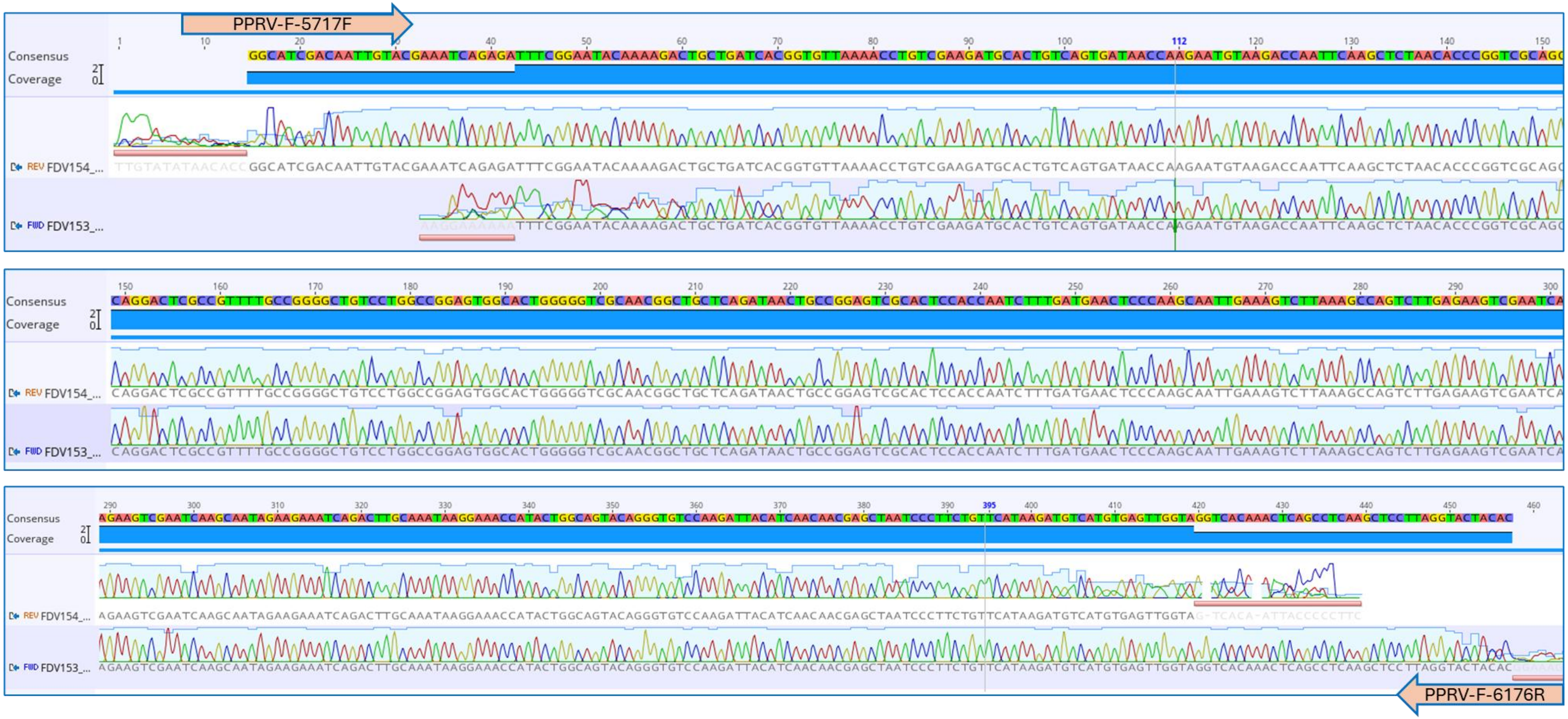

Figure S9. Primer combination PPRV-F-K9: Presentation of the chromatograms and the consensus sequence following the Sanger sequencing procedure. The positions of the primers used are indicated by arrows.

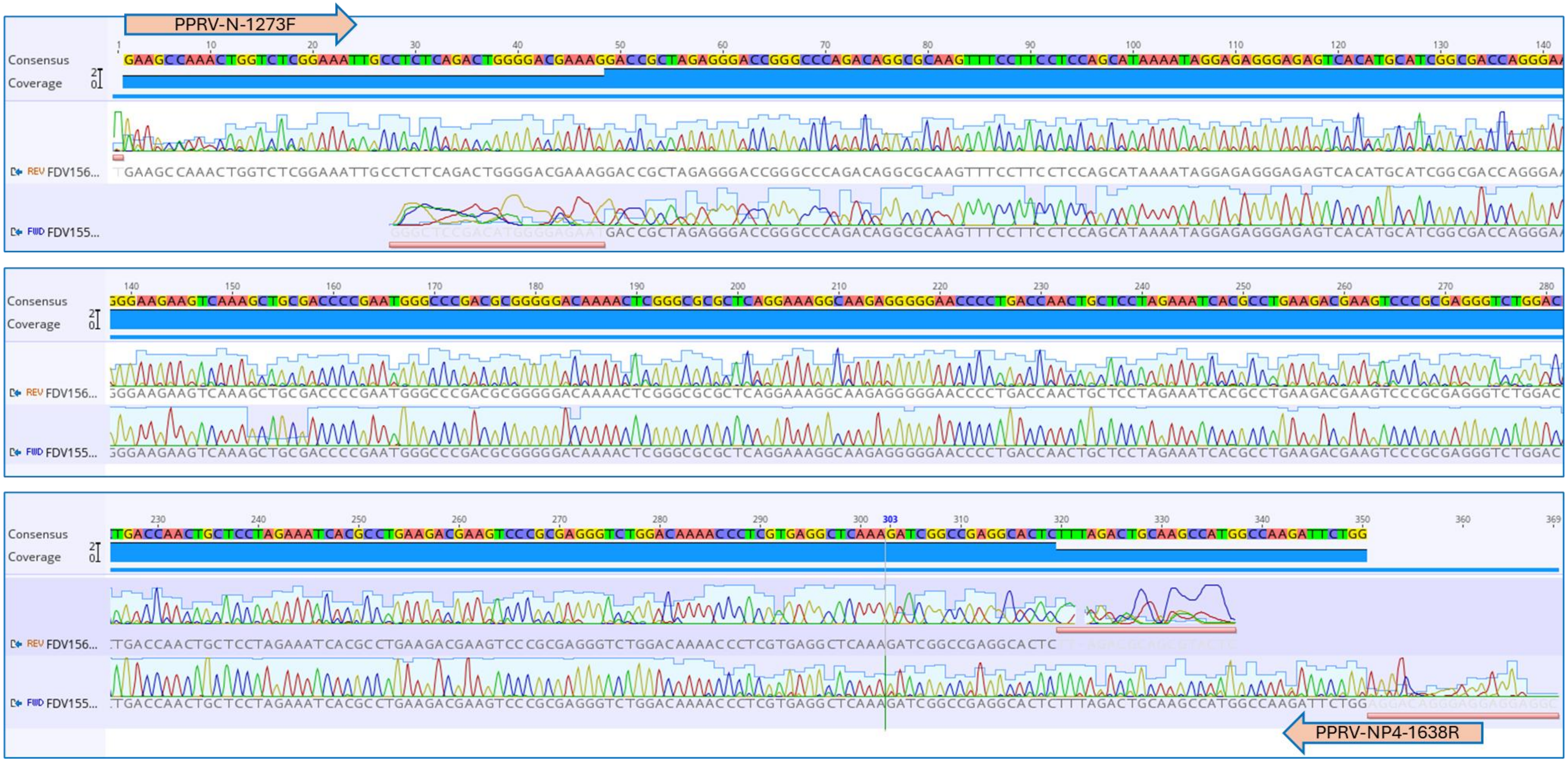

Figure S10. Primer combination PPRV-N-K3: Presentation of the chromatograms and the consensus sequence following the Sanger sequencing procedure. The positions of the primers used are indicated by arrows.

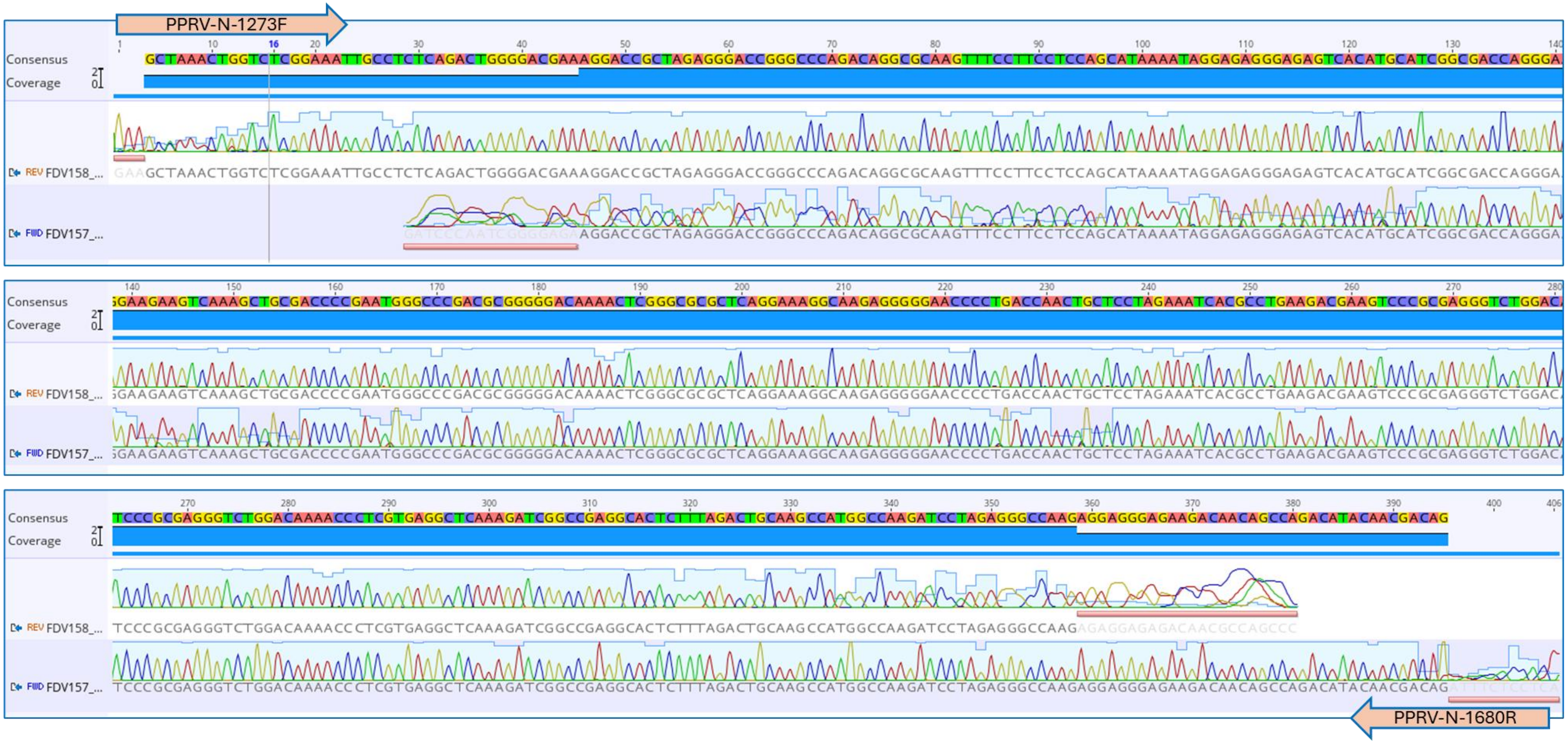

Figure S11. Primer combination PPRV-N-K15: Presentation of the chromatograms and the consensus sequence following the Sanger sequencing procedure. The positions of the primers used are indicated by arrows.
